# Endoscopic Submucosal Dissection and Chemoradiotherapy versus Esophagectomy for T1a-m3 or T1b Esophageal Squamous Cell Carcinoma: A Meta-analysis

**DOI:** 10.1055/a-2926-4315

**Published:** 2026-08-14

**Authors:** Jurandir B. da Cruz, Megui M. Gallegos, Vitor O. Brunaldi, Nelson T. Miyajima, Rayssa Campos, Silvia L. Calle, Evellin S. V. Valentim, Matheus de Oliveira Veras, Eduardo G. H. de Moura

**Affiliations:** 1 Gastrointestinal Endoscopy UnitDepartment of Gastroenterology 28133 Hospital das Clínicas (HCFMUSP), University of São Paulo School of MedicineSão PauloSão PauloBrazil

**Keywords:** endoscopy upper GI tract, precancerous conditions and cancerous lesions (dysplasia and cancer) stomach, endoscopic resection (ESD, EMRc, …), GI radiology, GI surgery

## Abstract

**Background and Study Aims** Esophagectomy is the standard treatment for superficial esophageal squamous cell carcinoma (ESCC) but carries substantial morbidity. Endoscopic submucosal dissection (ESD) followed by adjuvant chemoradiotherapy (CRT) is an organ-preserving alternative, but its oncologic efficacy in T1aM3 disease with lymphovascular invasion (LVI) and in T1b disease remains uncertain. We compared ESD+CRT with esophagectomy for these lesions.

**Patients and Methods** Medline, Embase, and Google Scholar were searched through June 2024 (PROSPERO CRD42024593581). Primary endpoints were the risk differences (RDs) between esophagectomy and ESD+CRT for 3-year disease-free survival (3y-DFS), 3-year overall survival (OS-3y), and 5-year OS (OS-5y); the secondary endpoint was serious adverse events (SAEs). Cumulative DFS and OS were estimated by Kaplan–Meier analysis and compared with the log-rank test.

**Results** Six retrospective studies (16 centers; 306 patients treated 2002–2021; ESD+CRT *n* = 170, esophagectomy *n* = 136) were included; mean follow-up was 49.8 and 53.1 months, respectively. Baseline age, LVI, and tumor depth were comparable. Three-year DFS was similar between groups (86.4% vs. 92.6%; RD 0.92, 95% CI 0.85–1.00), as was OS at 3 years (RD −0.02, 95% CI −0.08 to 0.45) and 5 years (RD −0.09, 95% CI −0.18 to 0.01). SAEs were significantly less frequent with ESD+CRT than with esophagectomy (RD 0.55, 95% CI 0.35–0.86; *p* = 0.009).

**Conclusions** In selected superficial ESCC, ESD+CRT achieved DFS and OS comparable to esophagectomy while significantly reducing SAEs. These retrospective data support ESD+CRT as a viable organ-preserving option, particularly for patients at high surgical risk.

## Introduction

Esophageal squamous cell carcinoma (ESCC) is the predominant histologic subtype of esophageal cancer, accounting for roughly 85% of cases worldwide and an estimated 513,485 new diagnoses each year. 1 The burden is projected to grow substantially: the International Agency for Research on Cancer (IARC) anticipates a 50% rise in esophageal cancer incidence between 2020 and 2040, approaching one million cases annually by 2040. 2 Within the superficial spectrum, early ESCC is confined to the muscularis mucosa (T1aM3N0M0), whereas superficial ESCC extends into the submucosa (T1b). Lesions limited to the lamina propria (Tis-T1aM2) seldom metastasize and are reliably cured by endoscopic resection alone. 3

**Fig. 1:**
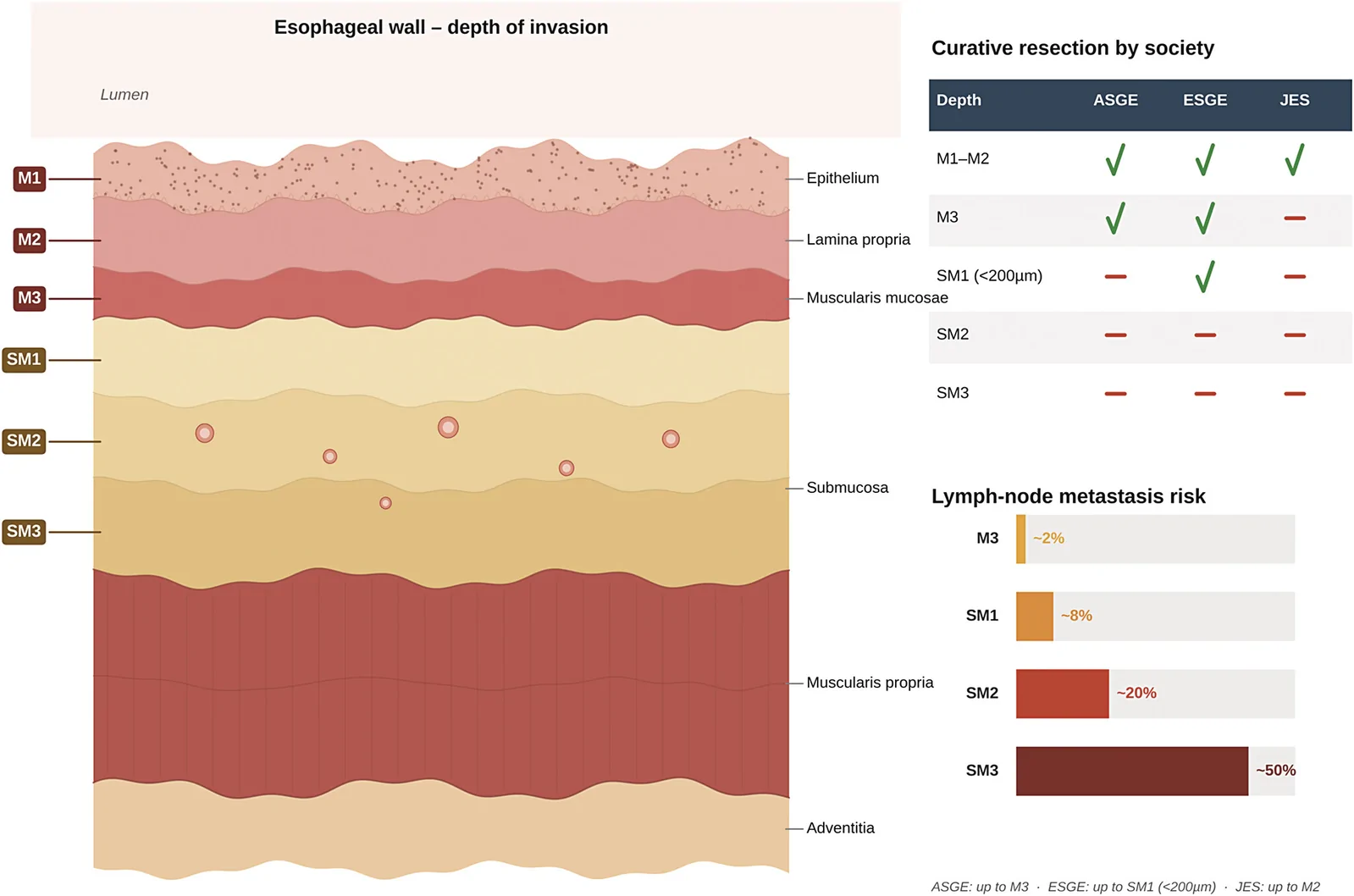
Illustration of esophageal wall layers with corresponding anatomic classifications (M1, M2, M3, SM1, SM2, SM3), society recommendations for curative ESD (ASGE, ESGE, JES), and associated lymphatic metastasis risks.

No consensus exists on the optimal adjuvant strategy for cT1aM3N0M0 without lymphovascular invasion (LVI) or for cT1bN0M0 managed with ESD, and international guidelines diverge in how they define curative resection and subsequent care. The American Society for Gastrointestinal Endoscopy (ASGE), drawing on five observational studies comparing endoscopic submucosal dissection (ESD) ( *n* = 463) and esophagectomy ( *n* = 495), regards ESD as curative for nonulcerated ESCC invading no deeper than the muscularis mucosa (T1aM3) without LVI, and imposes no restriction on lesion size. 4 The European Society of Gastrointestinal Endoscopy (ESGE), based on three retrospective studies comparing ESD ( *n* = 664) and esophagectomy ( *n* = 578), similarly considers ESD effective for noncircumferential lesions invading up to the upper third of the submucosa (<200 μm; T1b SM1) without LVI. 5 6 In contrast, the Japanese Esophageal Society (JES) – guided by lymph node metastasis risks of 8% for M3, 11% for SM1, 30% for SM2, and 61% for SM3 – restricts curative ESD to lesions confined to the lamina propria (T1aM2) and deems T1aM3 only relatively curable when LVI is absent and tumor features are favorable (well-differentiated, ≤3 cm, or ≤2 cm with ulceration) 7 ( Fig. 1 ).

Esophagectomy remains the reference treatment for stage I ESCC, achieving roughly 80% three-year survival, yet it carries substantial morbidity that durably impairs quality of life. 8 9 As an alternative, chemoradiotherapy (CRT) combining 5-fluorouracil (5-FU) with platinum agents has emerged as an organ-preserving strategy. 10 11 Radiotherapy targets both the primary tumor and the regional lymph nodes, while 5-FU and platinum agents disrupt DNA synthesis. 12 In the JCOG9708 phase II trial, CRT achieved a 90% complete response rate and a four-year overall survival (OS) of 80.5%; in a complementary analysis, 26 of 76 patients (34.2%) developed residual disease or locoregional recurrence without distant metastasis after CRT. 13

Tri-modal therapy combining ESD, radiotherapy, and chemotherapy offers a promising organ-preserving alternative to esophagectomy for superficial ESCC. 14 15 Yet the influence of ESD+CRT on recurrence and mortality in superficial ESCC – specifically T1aM3 with LVI and T1b disease – remains poorly defined. To address this gap, this systematic review and meta-analysis (SRMAs) quantified the risk differences (RDs) between esophagectomy and ESD+CRT for 3-year disease-free survival (DFS), 3y-OS, and 5-y OS. Cumulative OS and DFS were additionally estimated using the Kaplan–Meier approach, and crude differences between groups were assessed using the log-rank test. Secondary outcomes comprised serious adverse events (SAEs), recurrence, and mortality in patients with lymphovascular involvement, a critical adverse prognostic factor.

## Material and Methods

**Fig. 2:**
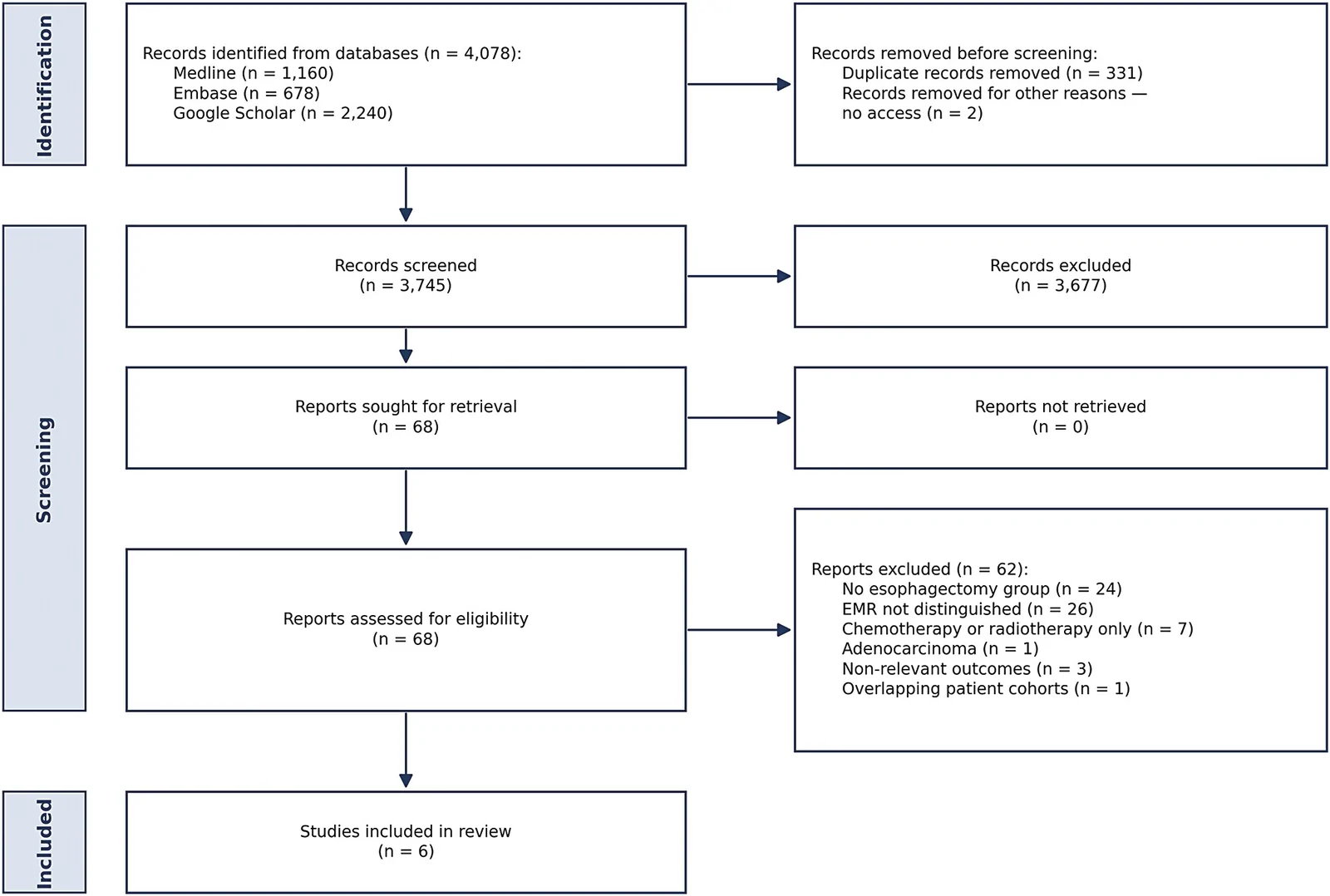
PRISMA 2020 flow diagram of study identification, screening, and inclusion. 16 A total of 4078 records were identified through database searching (Medline, *n* = 1,160; Embase, *n* = 678; Google Scholar, *n* = 2240). After removal of 331 duplicates and 2 records that could not be accessed, 3745 records were screened, of which 3677 were excluded by title and abstract. Of the 68 reports assessed for eligibility, 62 were excluded for the absence of an esophagectomy comparator group ( *n* = 24), failure to distinguish ESD from EMR ( *n* = 26), chemotherapy or radiotherapy only ( *n* = 7), adenocarcinoma histology ( *n* = 1), nonrelevant outcomes ( *n* = 3), and overlapping patient cohorts ( *n* = 1). Six studies met all criteria and were included in the systematic review and meta-analysis. *ESD* , endoscopic submucosal dissection; *EMR* , endoscopic mucosal resection. Adapted from Page MJ, McKenzie JE, Bossuyt PM, et al. The PRISMA 2020 statement: an updated guideline for reporting systematic reviews. *BMJ* 2021;372:n71.

This SRMA was conducted in accordance with the methodologic standards of the Cochrane Handbook for Systematic Reviews of Interventions and is reported following the Preferred Reporting Items for Systematic Reviews and Meta-Analyses (PRISMA) statement ( Fig. 2 ). 16

To promote transparency and avoid duplication, the protocol was prospectively registered with the International Prospective Register of Systematic Reviews (PROSPERO) 17 under registration number CRD42024593581.

We searched Medline, Embase, and Google Scholar for studies on the treatment of early esophageal cancer published through June 2024. Search terms combined the concepts esophagus cancer, endoscopic resection, CRT, and esophagectomy; the complete strategy is provided in the Supplementary Material ( **Supplementary Table S1** ).

Eligible studies were comparative analyses of esophagectomy versus ESD with adjuvant CRT for ESCC staged T1aM3 or T1b without clinical evidence of metastasis or nodal involvement. Studies using endoscopic resection other than ESD were excluded, as this SRMA specifically targeted the deeper T1aM3 and T1b lesions. We also excluded post-ESD regimens employing radiotherapy or chemotherapy alone, because both the JES and ESGE endorse combined CRT – or esophagectomy – as the preferred complementary treatment for superficial advanced ESCC.

Esophagectomies performed after oncologic staging by ESD were analyzed together with upfront esophagectomies, as esophagectomy constituted the definitive treatment in both settings and prior ESD altered neither the surgical technique nor the oncological risk.

The primary outcomes were the risk differences (RDs) between esophagectomy and ESD+CRT for 3-year disease-free survival (DFS-3y), 3y-OS, and 5y-OS. Cumulative DFS and OS were additionally estimated using the Kaplan–Meier approach; crude differences between groups were assessed using the log-rank test. The secondary outcome was the occurrence of SAEs. Because lymphovascular involvement is an established predictor of poor prognosis, recurrence and mortality were also examined specifically within this subgroup under CRT or esophagectomy.

Statistical analyses were performed in R (R Foundation for Statistical Computing; RStudio, R version 4.4.1) and Review Manager 5.4 (RevMan 5.4, Cochrane). 18 For each cohort, the epidemiologic profile and tumor characteristics were analyzed by disease stage using forest plots, box plots, and linear regression. Forest plots were used primarily to compare the primary outcomes between groups, providing a visual summary of effect sizes and their confidence intervals across studies.

Continuous and categoric variables were compared using the mean difference (MD) and RD, respectively. The RD reflects the rate or frequency of each variable, whereas the MD is derived from means and standard deviations (SDs). Statistical heterogeneity across studies was assessed with the chi-square (χ ^2^ ) and Higgins' ( *I* ^2^ ) tests, and a fixed-effects model was applied. A two-sided *p* -value below 0.05 was considered statistically significant, with effects reported alongside 95% confidence intervals (CIs).

When standard deviations for age or tumor size were not explicitly reported, they were estimated mathematically from the range between maximum and minimum values. 19 Functional status reported as Eastern Cooperative Oncology Group (ECOG) or American Society of Anesthesiologists (ASA) class was converted to the Charlson Comorbidity Index (CCI) using a previously validated approach 20 ; the CCI is anchored to one-year inpatient mortality data. 21 Esophagectomies performed after oncologic staging by ESD were analyzed together with upfront esophagectomies, as esophagectomy was the definitive treatment in both cases.

SAEs for esophagectomy and ESD+CRT were classified according to the US Food and Drug Administration (FDA) framework. 22 Toxicities in the CRT group were graded with the National Cancer Institute Common Terminology Criteria for Adverse Events, with emphasis on grade ≥3 events. 23

Risk of bias was assessed by both Cochrane Risk Of Bias In Non-randomized Studies of Interventions (ROBINS-I) ( **Supplementary Table S2** ) 24 and Newcastle–Ottawa Scale (NOS) for cohort studies 25 ( **Supplementary Table S3** ). ROBINS-I appraises seven domains – confounding, selection bias, classification of interventions, deviations from intended interventions, missing data, outcome measurement, and selective reporting – rating each domain, variable, and the study overall as low, moderate, serious, or critical risk. The NOS appraises three domains – selection, comparability, and outcome – awarding a maximum of 4, 2, and 3 stars, respectively, and these scores were mapped to the Agency for Healthcare Research and Quality (AHRQ) categories for interpretation.

Finally, the overall certainty of the extracted evidence was rated using the Grading of Recommendations Assessment, Development, and Evaluation (GRADE) framework ( https://www.gradepro.org/ ) ( **Supplementary Table S4** ). 26 The GRADEpro GDT is a tool that supports clinical guideline development and evidence quality assessment.

## Results

### Demographic and Clinicopathologic Features

Our search retrieved 3,745 records, of which 3,677 were excluded because the title or abstract did not meet the inclusion criteria. Of the 68 studies advanced to full-text review, 24 lacked an esophagectomy group, 26 did not distinguish ESD from EMR, 1 pooled adenocarcinoma with squamous cell carcinoma, and 7 reported adjuvant radiotherapy or chemotherapy alone; all were excluded. A further three studies were excluded because their datasets were unsuitable for the outcomes analyzed, and the study by Ikeda et al. 27 was excluded owing to potential patient overlap with the cohort of Koterazawa Yasufumi, as both groups share the same institutional affiliation. Six retrospective studies published between 2018 and 2024 ultimately met all criteria. 28 29 30 31 32 33

The pooled population comprised 306 patients with superficial ESCC treated between 2002 and 2021 across 16 specialized centers. Multidisciplinary teams delivered both treatments: ESD+CRT ( *n* = 170) and esophagectomy ( *n* = 136). Esophagectomy followed prior ESD in approximately 88% of surgical cases ( *n* = 121/136). Treatment allocation was driven chiefly by patient preference after a multidisciplinary discussion of the risks and benefits of each option. The demographic and oncological profiles of patients undergoing ESD+CRT or esophagectomy are summarized in Tables 1 and 2 , respectively. Mean follow-up was 49.8 months for ESD+CRT (median interval, 19 months) and 53.1 months for esophagectomy (median interval, 20 months).

**Table 1:** Characteristics of patients submitted to ESD+CRT.

Author	Number of Patients	Men	Women	Median Age	Median Horizontal Size (mm)	Upper Thorax	Middle Thorax	Lower Thorax	T1aM3	T1b	T1b SM1	T1b SM2	T1b SM3	LVI Invasion	Lymphatic Invasion	Vascular Invasion	R0 (vertical and horizontal margin)	Positive Vertical Margin	Positive Horizontal Margin
Koterazawa	31	24	7	68	29	3	20	5	3	28	18	10	0	14	11	3	25	4	2
Tanaka	30	32	1	65	22	4	21	8	0	33	9	24	0	16	13	9	30	1	2
Gen Suzuki	34	29	5	69	–	9	15	10	11	23	–	–	–	21	9	12	23	7	4
Jun Li	14	–	–	61.4	–	–	–	–	2	12	12	0	0	–	–	–	7	7	–
Xiao Li	42	–	–	63.3	29.9	–	–	–	–	–	–	–	–	–	–	–	–	–	
Zhu	19	14	5	64.4	31	3	8	8	0	19	6	13	0	2	–	–	7	6	6

Clinicopathologic characteristics of patients treated with ESD followed by adjuvant chemoradiotherapy (ESD+CRT). Data extracted from six retrospective studies published between 2018 and 2024. LVI: lymphovascular invasion; SM: submucosal; R0: complete resection with negative margins.

**Table 2:** Characteristics of patients submitted to esophagectomy.

Author	Number of Patients	Men	Women	Median Age	Median Horizontal Size (mm)	Upper Thorax	Middle Thorax	Lower Thorax	T1aM3	T1b	T1b SM1	T1b SM2	T1b SM3	LVI Invasion	Lymphatic Invasion	Vascular Invasion	Positive Lymph Nodes Detected at Surgery
Koterazawa	28	24	4	66	34	6	20	5	8	20	14	6	0	17	16	1	9
Tanaka	19	15	4	63	38	3	11	5	0	19	6	13	0	17	12	15	1
Gen Suzuki	26	24	2	65	–	6	15	5	8	18	–	–	–	21	13	8	–
Jun Li	25	–	–	61.4	–	–	–	–	1	19	19	0	0	–	–	–	2
Xiao Li	15	–	–	63.3	29.9	–	–	–	–	–	–	–	–	–	–	–	1
Zhu	2	22	1	63.1	42	2	9	12	0	23	7	9	0	4	–	–	0

Clinicopathologic characteristics of patients treated with esophagectomy (upfront or following noncurative ESD). Data extracted from six retrospective studies published between 2018 and 2024. LVI: lymphovascular invasion; SM: submucosal.

**Fig. 3:**
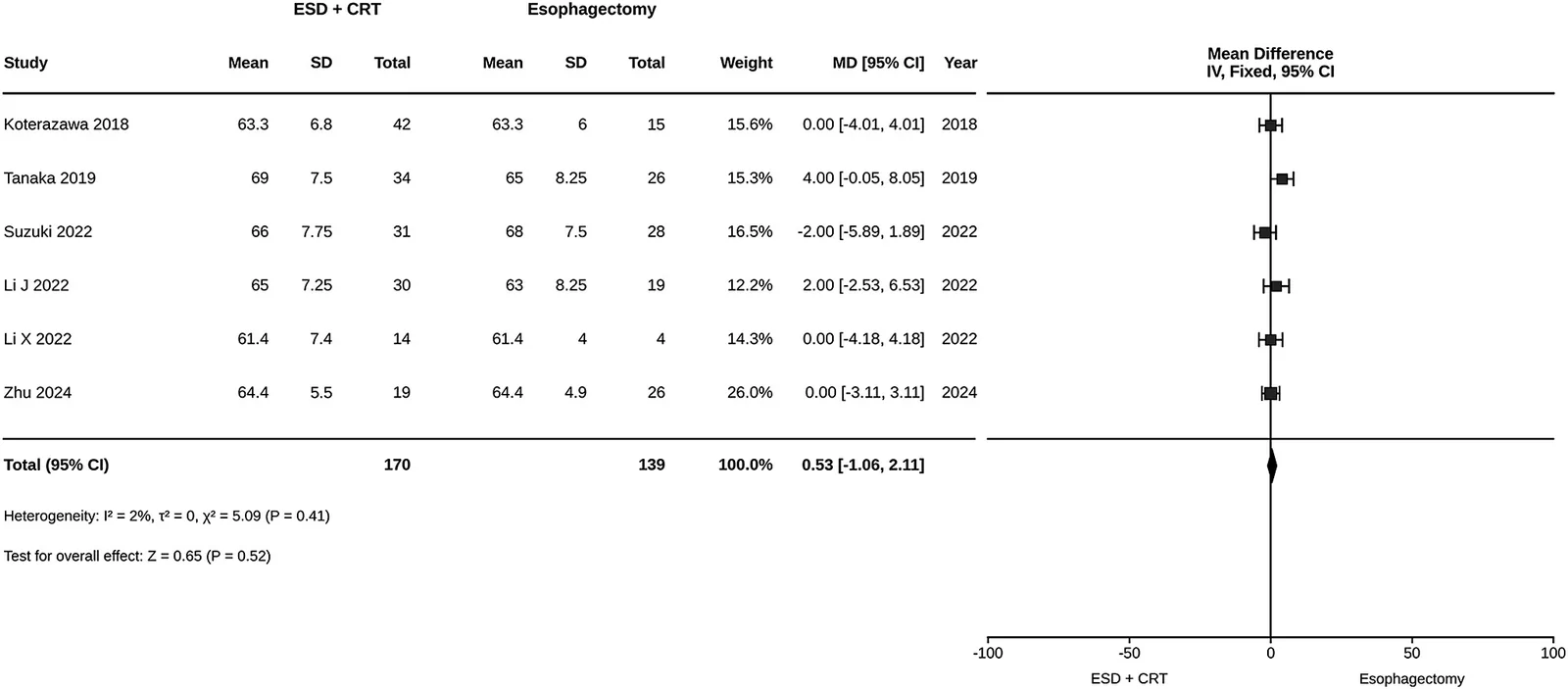
The data compare the mean age of patients undergoing ESD+CRT versus esophagectomy. The forest plot shows no significant difference in mean ages between the two groups, with an overall mean difference of MD = 0.53 years; 95% CI: −1.06 to 2.1.The heterogeneity is low ( *I* ^2^ = 2%, *p* = 0.41), indicating consistent results across studies.

Four studies comprising 173 patients (ESD+CRT = 86, esophagectomy = 87) reported functional status. The distribution of CCI categories did not differ significantly between groups. Mean age was 65.5 years (ESD+CRT) and 63.3 years (esophagectomy), with no significant difference between groups (MD = 0.53 years; 95% CI: −1.06 to 2.1) ( Fig. 3 ).

**Fig. 4:**
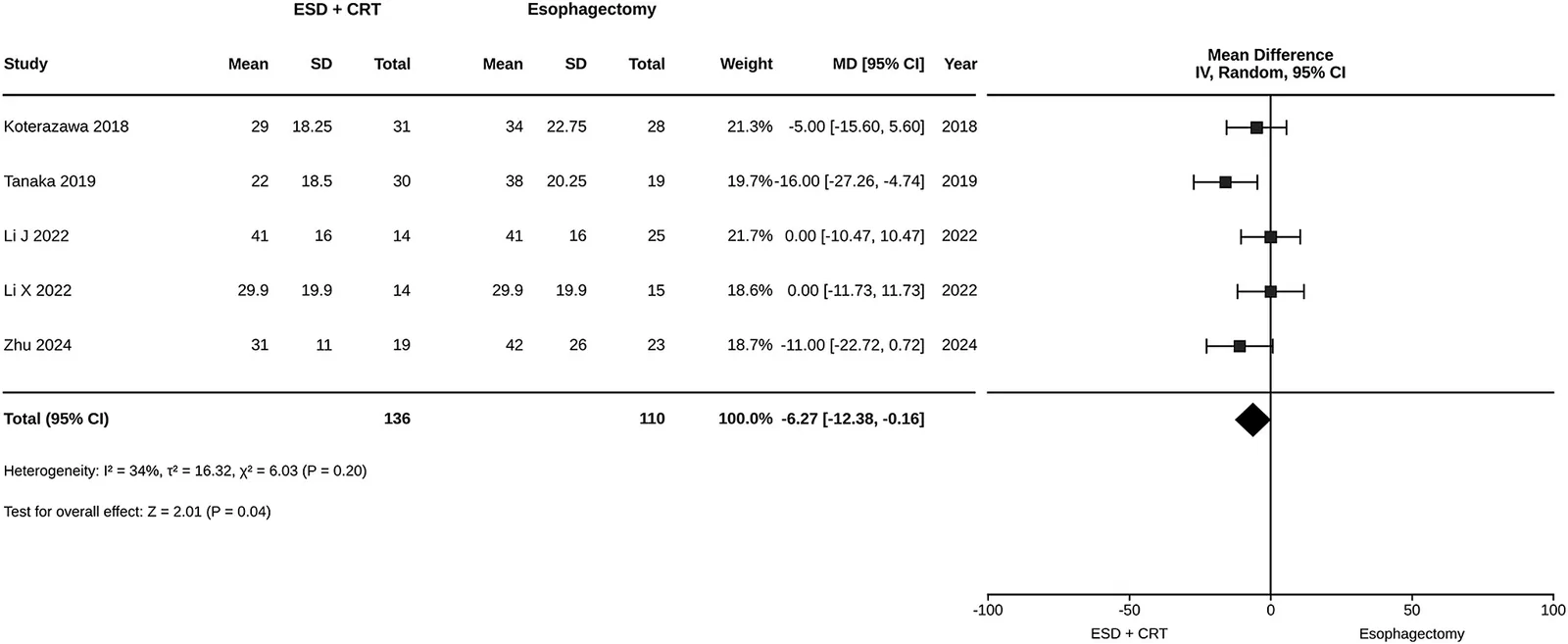
Analysis of four observational studies (2018-2024) comparing tumor sizes treated with ESD+CRT versus esophagectomy. The forest plot indicates that only the Tanaka study demonstrated statistically significant differences in tumor size. The pooled meta-analysis showed a significant difference in tumor size (MD = −6.27 mm; 95% CI: −12.38 to −0.16; *p* = 0.04)

The weighted mean tumor size was 27.9 mm for ESD+CRT and 35.9 mm for esophagectomy. The pooled meta-analysis demonstrated a statistically significant difference in tumor size between groups (MD = −6.27 mm; 95% CI: −12.38 to −0.16; *p* = 0.04) ( Fig. 4 ), driven primarily by the Tanaka study. In a complementary comparison of weighted group means, the difference did not reach statistical significance.

**Fig. 5:**
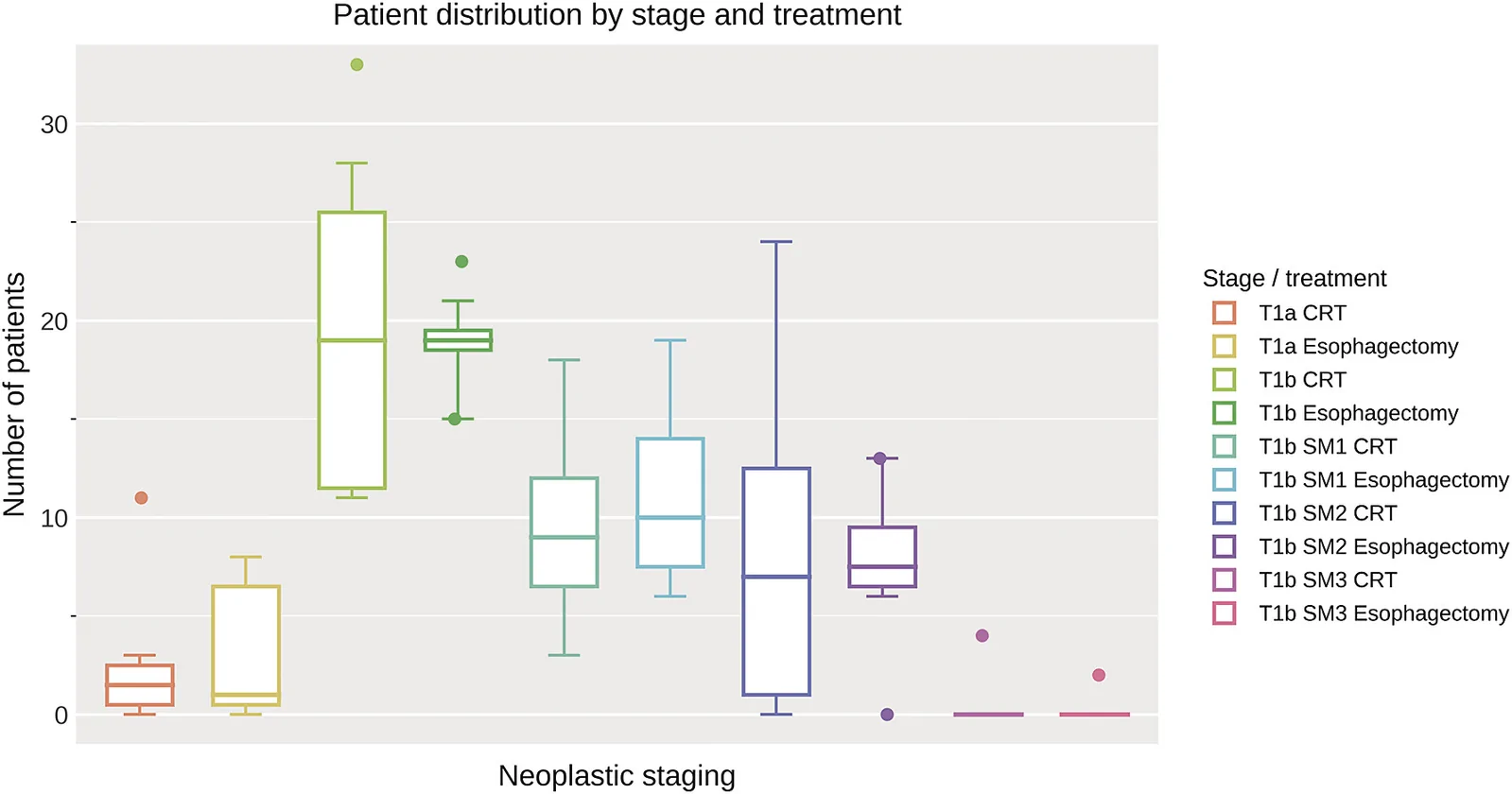
The box plot shows the distribution of patients across different neoplastic stages (T1a, T1b, T1b SM1, T1b SM2, T1b SM3) treated with ESD+CRT or esophagectomy. There is no clear or consistent difference in the number of patients treated with each modality across the stages, suggesting that the choice of treatment may depend on factors other than neoplastic staging, such as patient characteristics or clinical preferences.

Neoplastic staging showed that approximately 90% of ESD+CRT lesions were T1b (38% SM1, 40.5% SM2, 3% SM3), as were 87% of esophagectomy lesions (46.5% SM1, 29.8% SM2, 1.75% SM3) ( Fig. 5 ). LVI was observed in 53/114 (46.5%) of the ESD+CRT cohort and 59/96 (61.4%) of the esophagectomy group, with no statistically significant difference between the groups.

### Treatment Outcomes and Supplementary Analyses

**Fig. 6:**
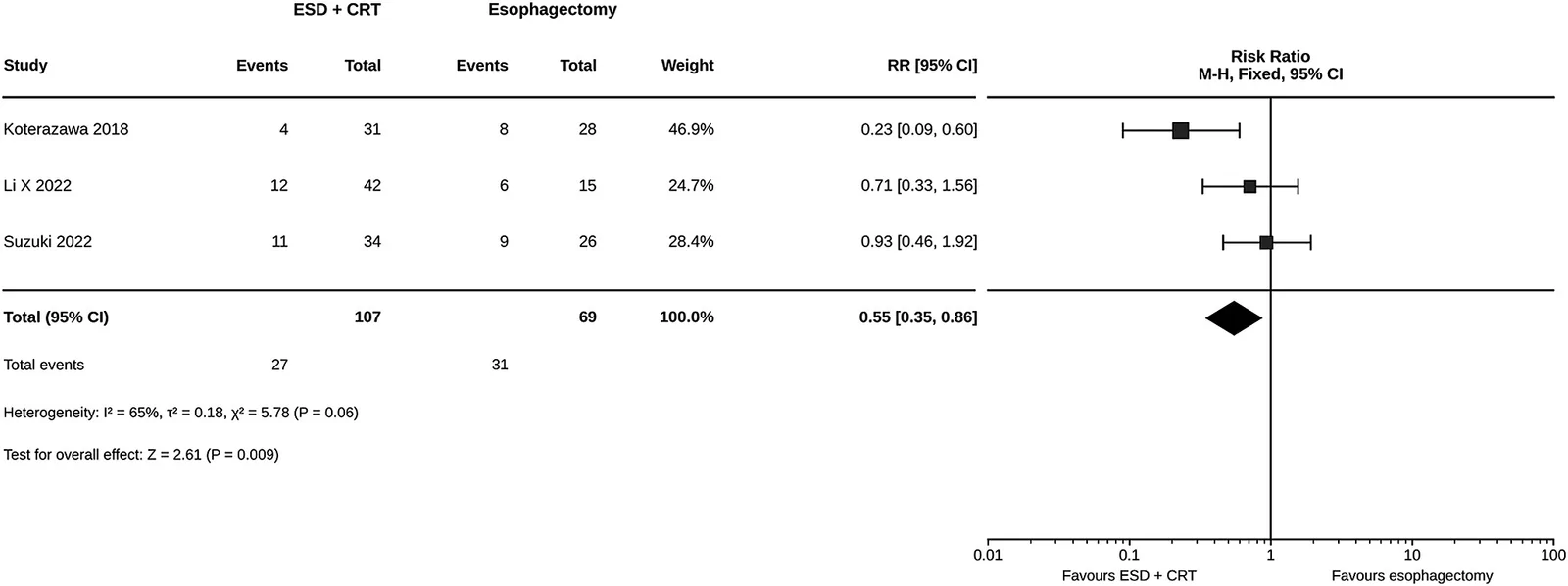
The forest plot compares SAEs between ESD+CRT and esophagectomy across four studies. The pooled analysis shows a statistically significant overall effect favoring ESD+CRT, with a risk difference of 0.55 (95% CI: 0.35 to 0.86, *p* = 0.009). While individual study results vary slightly, the overall data suggest that ESD+CRT is associated with a lower rate of SAEs compared to esophagectomy.

The most frequently reported grade ≥3 adverse events attributable to CRT were hematologic toxicity (12/107, 11%) and esophagitis (4/107, 3%); no grade 4 SAEs were associated with CRT. Stenosis (7/59, 11%) was the most common SAE after ESD. In the esophagectomy cohort, the leading SAEs were anastomotic leakage (9/69, 13%), respiratory complications (12/69, 17.4%), and nerve palsy (7/69, 10%). SAEs were significantly more frequent after esophagectomy than after ESD+CRT (RD = 0.55; 95% CI: 0.35–0.86; *p* = 0.009) ( Fig. 6 ). Treatment-related mortality showed no differences (RD = 0.23; 95% CI [0.05 to 1.03]).

At three years, 158 of 170 patients (mean, 92.9%; 95% CI: 85.7–100%) in the ESD+CRT group and 128 of 136 (mean, 94.1%; 95% CI: 86.6–100%) in the esophagectomy group were alive. Four studies totaling 204 patients (ESD+CRT = 117, esophagectomy = 87) reported five-year OS. At five years, 100 of the 117 ESD+CRT patient group (mean: 85.4%, 95% CI: 51.4–100%) and 79 of the 87 esophagectomy patients (mean, 90.8%; 95% CI: 89.2–100%) were alive.

**Fig. 7:**
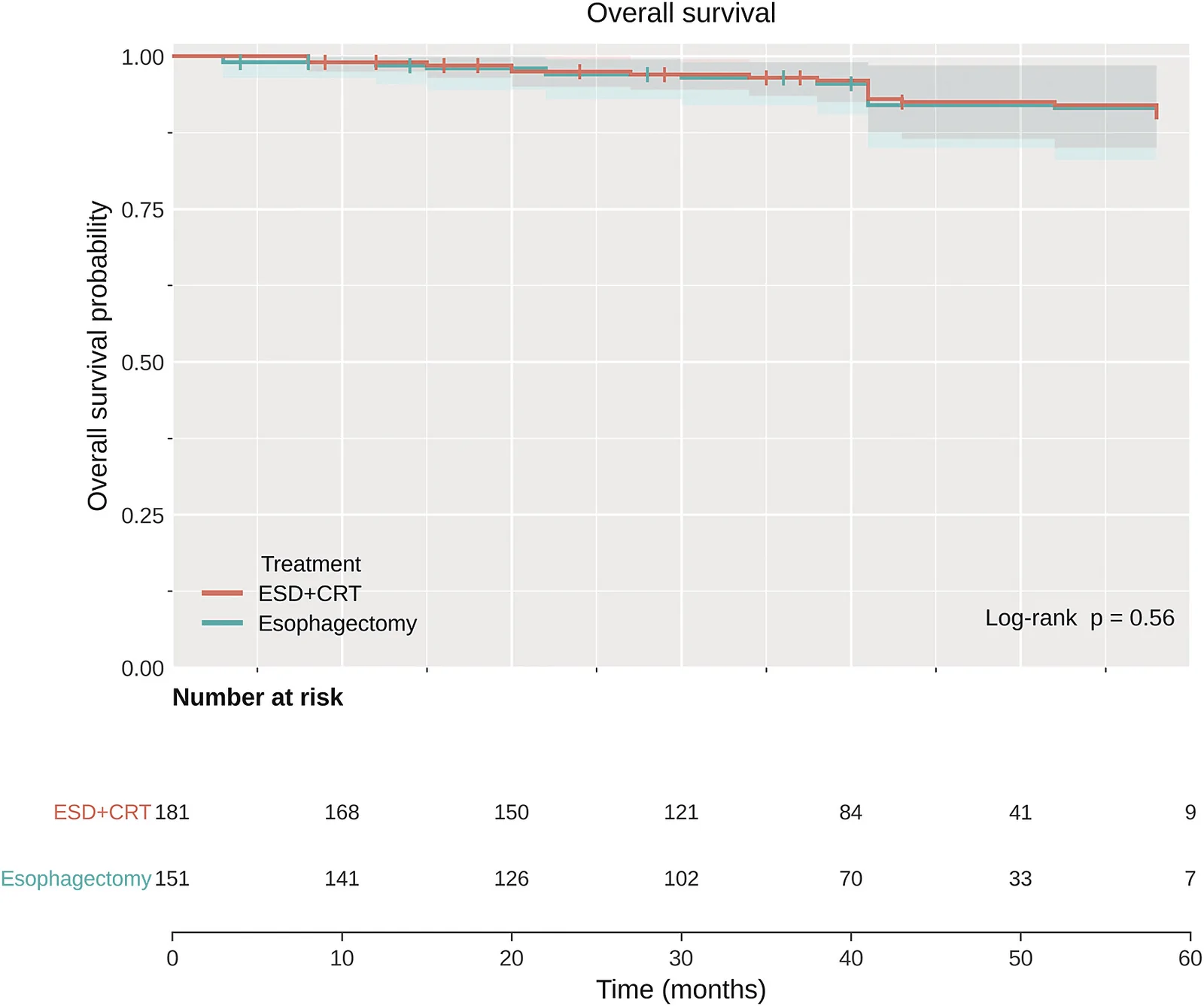
Kaplan–Meier estimates showed no statistically significant difference in cumulative overall survival between groups (log-rank test).

**Fig. 8:**
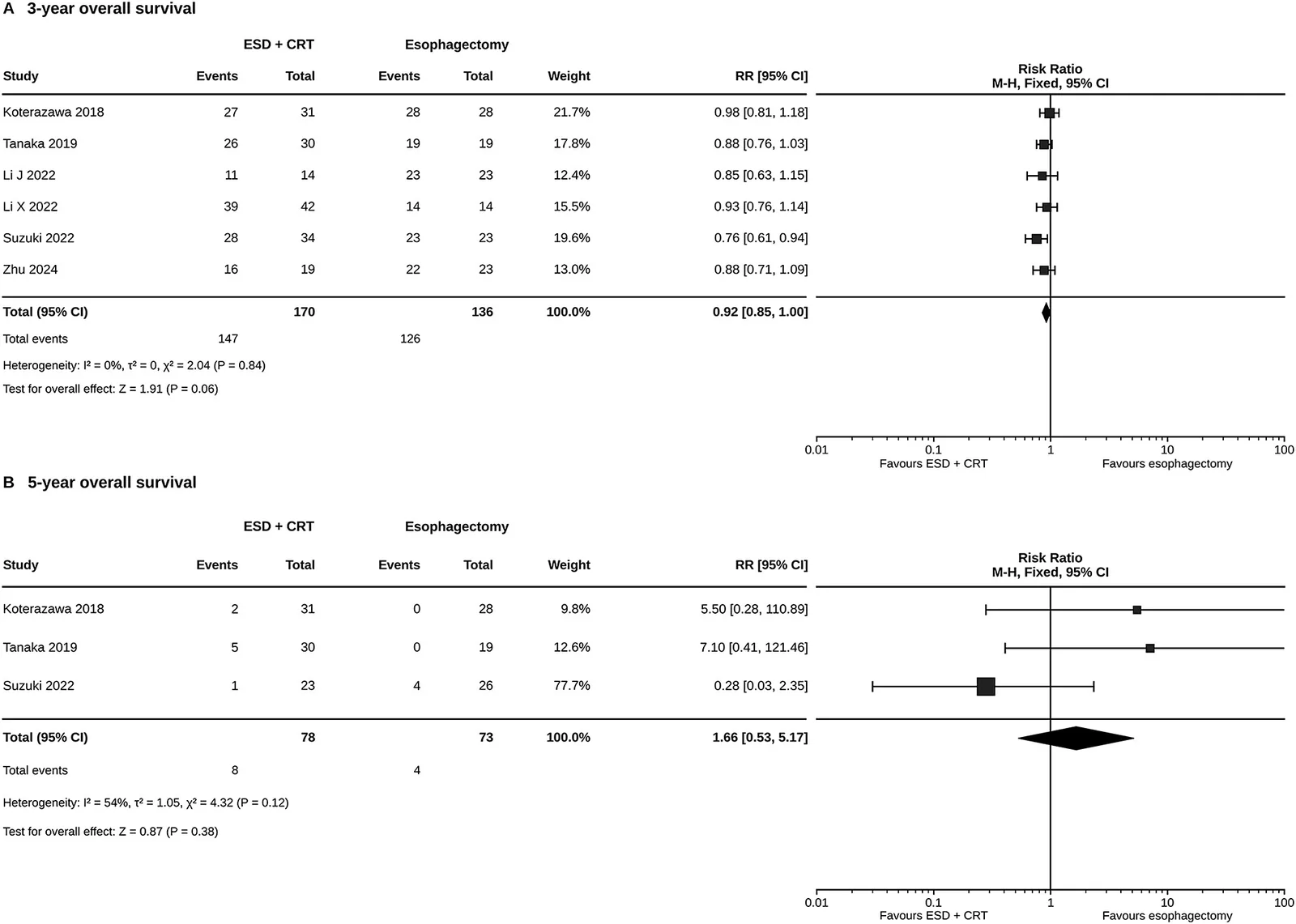
The upper graph compares the risk-difference of 3-year overall survival for ESD+CRT and esophagectomy. It shows a pooled risk difference of –0.02 (95% CI: -0.08 to 0.45), indicating no statistically significant difference between the two groups, with low heterogeneity ( *I* ^2^ = 0%). The 5-year overall survival graph (lower) shows a pooled risk difference of –0.09 (95% CI: –0.18 to 0.01). Overall, both treatments demonstrate comparable survival outcomes.

Cumulative OS was estimated using the Kaplan–Meier method. The log-rank test revealed no significant difference in OS between the ESD+CRT and esophagectomy groups ( Fig. 7 ). The RD for 3-y OS was −0.02 (95% CI: −0.08 to 0.45) ( Fig. 8 ), and for 5-y OS, the RD was −0.09 (95% CI: −0.18 to 0.01) ( Fig. 8 ).

The ESD+CRT group experienced 29 recurrences among 170 patients (mean, 17%; 95% CI: 51.4–92.8) – 9 local, 11 nodal, and 9 metastatic – with shortest mean times to recurrence of 11.2, 15.4, and 25.5 months after ESD, respectively. The esophagectomy group experienced 8 recurrences among 136 patients (mean, 5.88%; 95% CI: 80.7–100%) – 1 peri-anastomotic, 2 nodal, and 5 metastatic – with shortest times to recurrence of 35, 24, and 29 months, respectively.

**Fig. 9:**
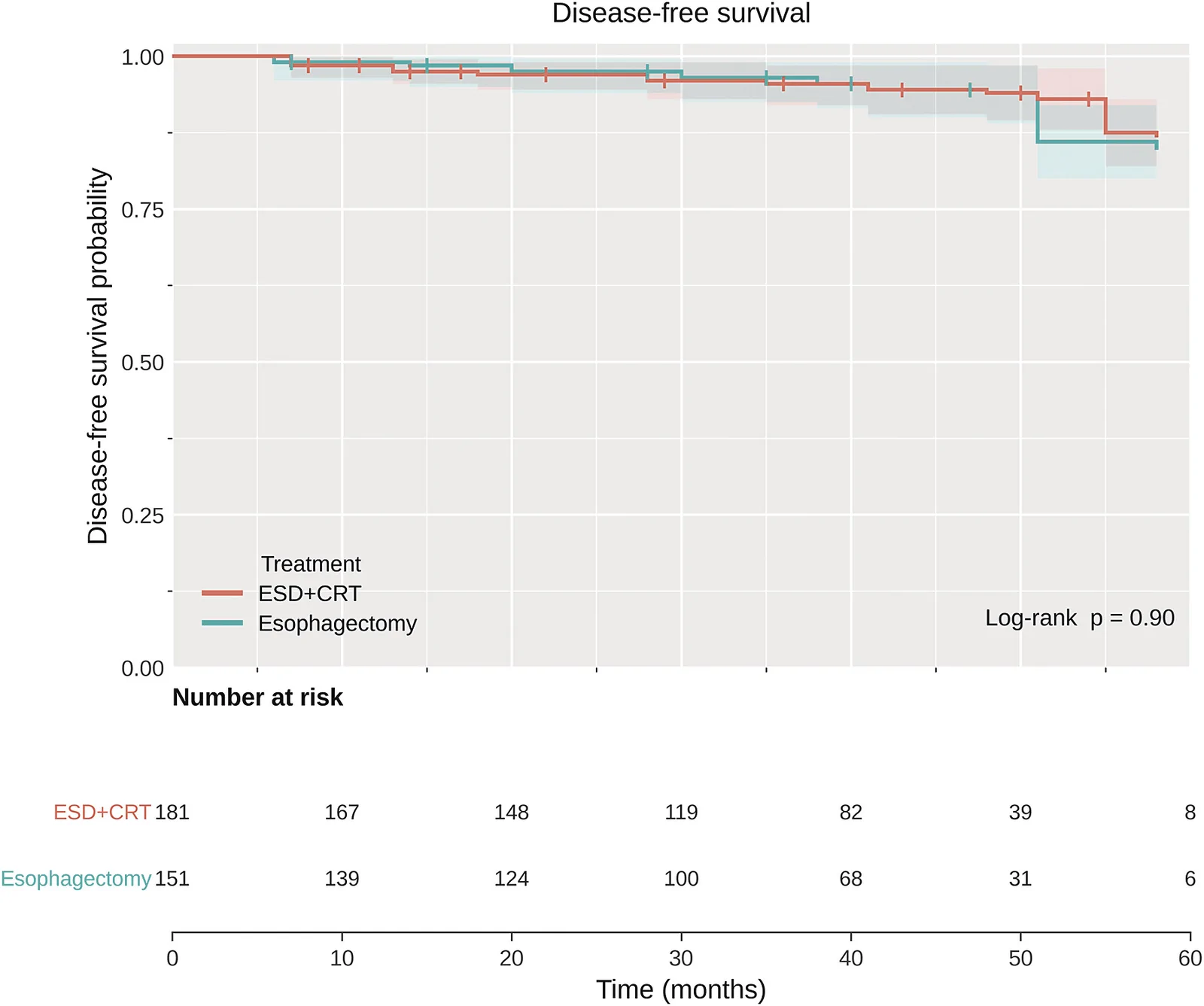
The Kaplan–Meier plots illustrate the disease-free survival for ESD+CRT and esophagectomy. The log-rank test shows no statistically significant difference in DFS between the groups.

The 3y-DFS was 86.4% (95% CI: 71.4–92.8%) for ESD+CRT and 92.6% (95% CI: 88.4–100%) for esophagectomy. DFS was estimated using the Kaplan–Meier method ( Fig. 9 ). The log-rank test showed no statistically significant difference in DFS between the groups ( Fig. 9 ).

**Fig. 10:**
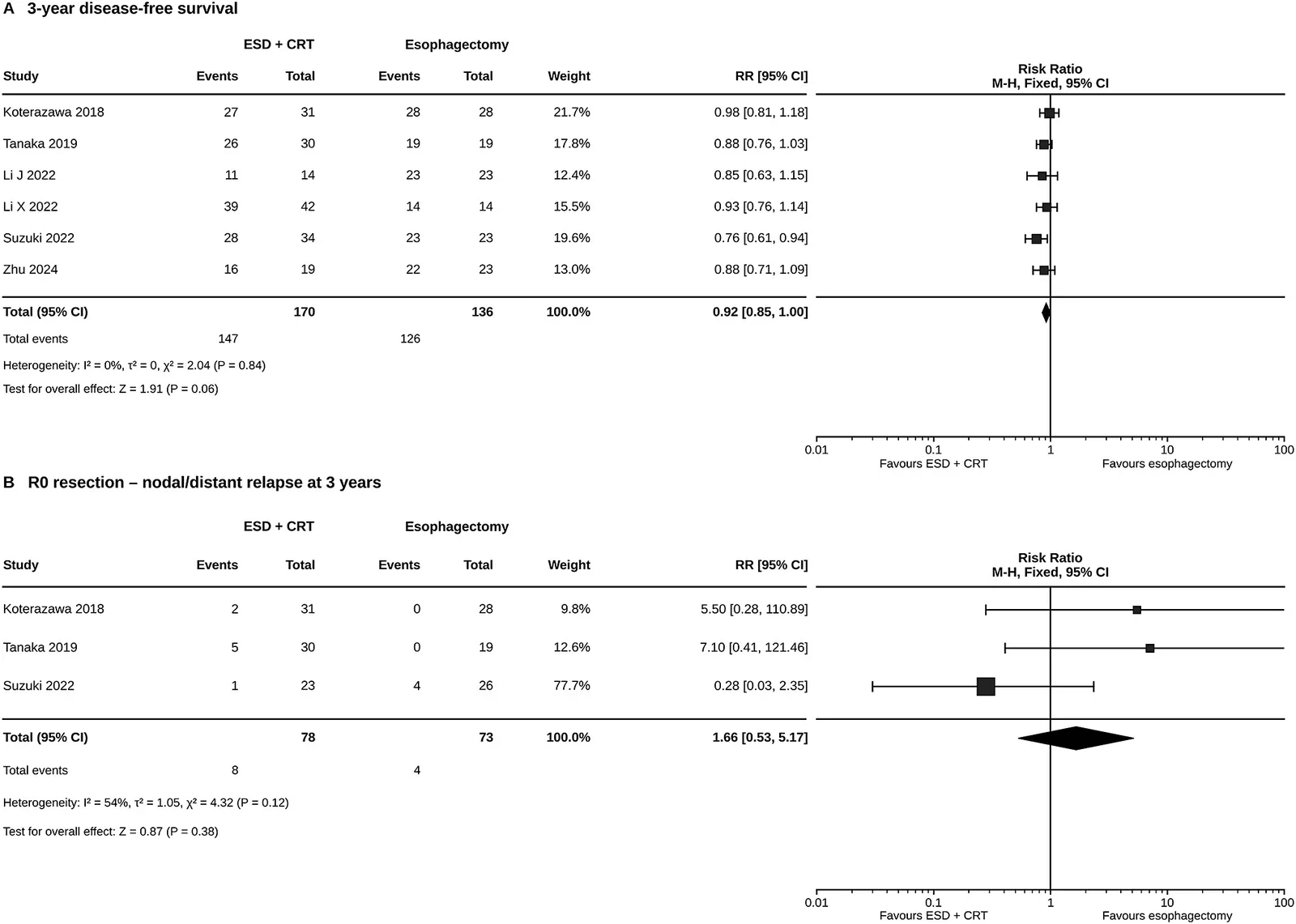
The upper graph shows no risk-difference in 3-year disease-free survival (RD: 0.92, 95% CI: 0.85–1). Heterogeneity is low ( *I* ^2^ = 0%), reflecting consistency across studies. The lower graph represents patients with R0 resection who present nodal and/or distant relapses in 3 years.

The risk difference (RD) for 3-year DFS between ESD+CRT and esophagectomy was 0.92 (95% CI: 0.85–1.00). A subgroup analysis confined to nodal or distant relapse after R0 resection likewise showed no significant difference between esophagectomy and ESD+CRT (RD = 1.55; 95% CI: 0.51–4.75) ( Fig. 10 ).

## Discussion

Baseline demographic characteristics (including age and functional status) and pathologic features (tumor size, LVI, and depth of invasion) did not significantly differ between the ESD+CRT group and the combined upfront-esophagectomy or post-ESD esophagectomy group. This balance indicates that the patient populations were comparable, strengthening the validity of the subsequent treatment–outcome comparisons.

Esophagectomy is the standard of care for superficial ESCC that cannot be managed endoscopically 34 35 ; however, it carries a high burden of SAEs that materially affects health and quality of life. Indeed, quality of life has become a central consideration after treatment of superficial esophageal cancer. 36 37 In a series of 486 patients who underwent esophagectomy, roughly 44.4% reported long-term sequelae, 38 and a comparative analysis of quality of life after esophagectomy ( *n* = 241) versus ESD ( *n* = 241) demonstrated significantly better treatment-related quality of life in the ESD group.

Our SRMA found comparable risk differences for DFS-3y, OS-3y, and OS-5y between patients treated with ESD+CRT and those treated with esophagectomy. Consistently, Kaplan–Meier analysis and log-rank tests did not reveal significant differences in OS or DFS between groups. Suzuki et al. 30 compared CRT with esophagectomy after noncurative ESD for cT1N0M0 disease and reported four-year OS of 84% versus 92%, respectively, without a significant difference ( *p* = 0.87). Notably, the esophagectomy arm of that study showed an unusual recurrence pattern: one nodal recurrence at 36 months, one peri-anastomotic recurrence at 35 months, and three metastases, the earliest at 29 months.

Ikeda et al. 27 reported that only 1 of 9 patients treated with ESD+CRT after an R0 resection experienced recurrence, whereas every patient with an R1 resection relapsed. Both groups received comparable doses of cisplatin and 5-FU, but radiotherapy differed: patients with negative endoscopic margins received 41.4 Gy in 23 fractions over five weeks (five days per week), whereas those with positive margins or invasion beyond SM3 received 50.4 Gy. These observations suggest that, although CRT effectively addresses cancer within the lymphatic system, it is less able to control residual disease at the ESD resection site.

CRT after ESD follows two distinct dose strategies: 41.4 Gy as prophylactic irradiation when resection margins are negative, and 50.4 Gy as definitive treatment when margins are positive. 39 Table 3 summarizes the various CRT doses and regimens used across the retrospective studies in our SRMA. Notably, there is significant heterogeneity among the regimens. The ongoing Japanese phase III randomized controlled ARMADILLO trial (JCOG1904) compares 50.4 Gy (with lymph node irradiation) against 60 Gy (without lymph node irradiation) in cT1b patients who did not undergo ESD; both are considered definitive doses. Given this variability, JCOG1904 may help establish the appropriate post-ESD radiation dose according to pathologic findings such as positive margins and/or LVI. 40 41

**Table 3:** Summary of Different Doses and Regimens of Chemoradiotherapy for R0 and R1 resections.

Author	Chemotherapy Regimen	RT R0	RT R1
Koterazawa	Cisplatin 70 mg/m ^2^ on days 1 and 29; Fluorouracil 700 mg/m ^2^ continuously from days 29 to 32.	41.4 Gy	50.4 Gy
Tanaka	Chemotherapy included: - FP (1000/100): 5-FU 1000 mg/m ^2^ /day (days 1–4, 29–32); CDDP 100 mg/m ^2^ (days 1, 29). - FP (800/80): 5-FU 800 mg/m ^2^ /day (days 1–5, 29–32); CDDP 80 mg/m ^2^ (days 1, 29). - FP (700/70): 5-FU 700 mg/m ^2^ /day (days 1–4, 29–32); CDDP 70 mg/m ^2^ (days 1, 29). Low-dose regimens for frail patients before 2010: 5-FU 200 mg/m ^2^ /day for 5 days; CDDP 4 mg/m ^2^ before radiotherapy; Docetaxel 10 mg/m ^2^ weekly.	40 Gy	>50 Gy
Gen Suzuki	Continuous 5-Fluorouracil 1000 mg/m ^2^ (days 1–4, 29–32) and cisplatin 75 mg/m ^2^ (days 1, 29).	40 Gy	50 Gy
Jun Li	Cisplatin 75 mg/m ^2^ +5-FU 1000 mg/m ^2^ /day for 4 days (R1 resection). Only 7 cases received 60 Gy.	60 Gy	60 Gy
Xiao Li	Not specified.	50–60 Gy	Not specified
Zhu	5-Fluorouracil+Cisplatin.	40 Gy	>50 Gy

Summary of chemoradiotherapy regimens and radiation doses used in the included studies, stratified by resection margin status (R0 vs. R1). 5-FU: 5-fluorouracil; CDDP: cisplatin; RT: radiotherapy; Gy: Gray.

In summary, our SRMA found no significant differences in OS or recurrence between ESD+CRT and esophagectomy. A focused analysis of R0 resections reinforced the absence of a significant difference in nodal and/or distant relapse between the two strategies. Moreover, although individual studies varied, the aggregate evidence indicates that ESD+CRT carries a significantly lower rate of SAEs than esophagectomy.

## Limitations

Our SRMA included only six retrospective studies, and this scarcity inherently heightens the risks of selection bias and incomplete data capture intrinsic to nonprospective designs. To limit confounding from histopathologic heterogeneity, we excluded studies that did not explicitly separate squamous cell carcinoma (SCC) from adenocarcinoma in their analyses.

To isolate the therapeutic effect of multimodal adjuvant regimens, we further restricted the analysis to studies that prospectively defined and compared chemotherapy with radiotherapy-only protocols. Although methodologically necessary to preserve internal validity, these stringent criteria markedly reduced the final cohort, limiting the generalizability of our findings and warranting caution when extrapolating to broader populations.

The restriction of included studies to Asian centers introduces potential epidemiologic bias. Japan’s national gastric cancer screening programs, for example, facilitate early detection and minimally invasive treatment to a degree that may not be reproduced in regions with less structured programs. In addition, the concentration of high-volume tertiary centers staffed by endoscopists with advanced ESD expertise likely influences procedural outcomes such as R0 resection rates and procedure-related adverse events. This regional variability in both screening infrastructure and technical proficiency may limit the generalizability of our findings to populations with different health care access or endoscopic training.

The retrospective design also constrains our ability to identify the factors driving the higher local recurrence observed in the ESD+CRT cohort. Subgroup analyses of R0 resection status and LVI were further limited by small sample sizes, inconsistent data granularity, and heterogeneous reporting standards, all of which reduce the statistical power to establish robust associations.

Finally, despite adjustment for variables such as age and functional status, our reliance on data pooled from multiple heterogeneous samples – rather than a single unified population – raises the possibility of unevenly distributed background characteristics. Such unmeasured confounders may have contributed to the observed between-group differences and cannot be fully excluded. This limitation underscores the need for prospective studies to better account for these factors and to provide a more comprehensive and reliable understanding of the outcomes.

## Conclusion

Our findings indicate that ESD with adjuvant CRT may be a viable and effective option for patients with T1aM3 ESCC and LVI, as well as for those with T1b disease. By combining ESD with adjuvant CRT, this strategy offers a promising organ-preserving alternative to esophagectomy for superficial ESCC, particularly for patients at elevated surgical risk.

This tri-modal approach appears to balance oncologic radicality with functional preservation, delivering OS comparable to that of esophagectomy. Nevertheless, the absence of well-defined criteria for stratifying recurrence risk currently limits the ability to recommend ESD with adjuvant CRT as a first-line treatment for T1aM3 ESCC with LVI or T1b disease.

## Supplementary Material

Ergänzendes Material Supplementary Material

## References

[1] Liu C Q Ma Y L Qin Q Epidemiology of Esophageal cancer in 2020 and projections to 2030 and 2040 Thoracic Cancer 2023 14 01 3 11 10.1111/1759-7714.14745 36482832 PMC9807450

[2] Morgan E Soerjomataram I Rumgay H The global landscape of Esophageal squamous cell carcinoma and Esophageal adenocarcinoma incidence and mortality in 2020 and projections to 2040: new estimates from GLOBOCAN 2020 Gastroenterology 2022 163 03 649 65800 10.1053/j.gastro.2022.05.054 35671803

[3] Wang J Zhang X Gan T Rao N N Deng K Yang J L Risk factors and a predictive nomogram for lymph node metastasis in superficial Esophageal squamous cell carcinoma World J Gastroenterol 2023 29 47 6138 6147 10.3748/wjg.v29.i47.6138 38186680 PMC10768412

[4] Al-Haddad M A Elhanafi S E Forbes N American Society for Gastrointestinal Endoscopy guideline on endoscopic submucosal dissection for the Management of Early Esophageal and Gastric Cancers: methodology and review of evidence Gastrointest Endosc 2023 98 03 285 3.05E40 10.1016/j.gie.2023.03.030 37498265

[5] Pimentel-Nunes P Libânio D Bastiaansen B AJ Endoscopic submucosal dissection for superficial gastrointestinal lesions: European Society of Gastrointestinal Endoscopy (ESGE) guideline - update 2022 Endoscopy 2022 54 06 591 622 10.1055/a-1811-7025 35523224

[6] Pimentel-Nunes P Dinis-Ribeiro M Ponchon T Endoscopic submucosal dissection: European Society of Gastrointestinal Endoscopy (ESGE) guideline Endoscopy 2015 47 09 829 854 10.1055/s-0034-1392882 26317585

[7] Kitagawa Y Ishihara R Ishikawa H Esophageal cancer practice guidelines 2022 edited by the Japan Esophageal society: part 1 Esophagus 2023 20 03 343 372 10.1007/s10388-023-00993-2 36933136 PMC10024303

[8] Pennathur A Farkas A Krasinskas A M Esophagectomy for T1 Esophageal cancer: outcomes in 100 patients and implications for endoscopic therapy Ann Thorac Surg 2009 87 04 1048 1055 10.1016/j.athoracsur.2008.12.060 19324126 PMC2912110

[9] Katz A Nevo Y Ramírez García Luna J L Long-term quality of life after Esophagectomy for Esophageal cancer Ann Thorac Surg 2023 115 01 200 208 10.1016/j.athoracsur.2022.07.029 35926638

[10] Tasaki Y Yamazaki T Miyazaki S Additional Chemoradiotherapy for superficial Esophageal squamous cell carcinoma after near-circumferential or full-circumferential non-curative endoscopic submucosal dissection: a retrospective study BMC Gastroenterol 2024 24 01 232 10.1186/s12876-024-03328-2 39044174 PMC11267816

[11] Suzuki G Yamazaki H Aibe N Endoscopic submucosal dissection followed by Chemoradiotherapy for superficial Esophageal cancer: choice of new approach Radiat Oncol 2018 13 01 246 10.1186/s13014-018-1195-7 30547811 PMC6295044

[12] Liu Y P Zheng C C Huang Y N He M L Xu W W Li B Molecular mechanisms of chemo- and radiotherapy resistance and the potential implications for cancer treatment MedComm 2021 2 03 315 340 10.1002/mco2.55 34766149 PMC8554658

[13] Kato H Sato A Fukuda H A phase II trial of Chemoradiotherapy for stage I Esophageal squamous cell carcinoma: Japan clinical oncology group study (JCOG9708) Jpn J Clin Oncol 2009 39 10 638 643 10.1093/jjco/hyp069 19549720

[14] Lu H Bei Y Wang C A retrospective cohort study to observe the efficacy and safety of endoscopic submucosal dissection (ESD) with adjuvant radiotherapy for T1a-MM/T1b-SM Esophageal squamous cell carcinoma (ESCC) PLoS One 2024 19 02 e0298792 10.1371/journal.pone.0298792 38386660 PMC10883569

[15] Mochizuki Y Saito Y Tsujikawa T Fujiyama Y Andoh A Combination of endoscopic submucosal dissection and Chemoradiation therapy for superficial Esophageal squamous cell carcinoma with submucosal invasion Exp Ther Med 2011 2 06 1065 1068 10.3892/etm.2011.319 22977621 PMC3440818

[16] Page M J McKenzie J E Bossuyt P M The PRISMA 2020 statement: an updated guideline for reporting systematic reviews Syst Rev 2021 10 01 89 10.1186/s13643-021-01626-4 33781348 PMC8008539

[17] Schiavo J H PROSPERO: an international register of systematic review protocols Med Ref Serv Q 2019 38 02 171 180 10.1080/02763869.2019.1588072 31173570

[18] Higgins J PT Thomas J Chandler JCochrane Handbook for Systematic Reviews of Interventions. Version 6.5. Cochrane2024

[19] Hozo S P Djulbegovic B Hozo I Estimating the mean and variance from the median, range, and the size of a sample BMC Med Res Methodol 2005 5 01 13 10.1186/1471-2288-5-13 15840177 PMC1097734

[20] Dobbins T A Badgery-Parker T Currow D C Young J M Assessing measures of comorbidity and functional status for risk adjustment to compare hospital performance for colorectal cancer surgery: a retrospective data-linkage study BMC Med Inf Decis Mak 2015 15 01 55 10.1186/s12911-015-0175-1 PMC4502567 26174550

[21] Jørgensen T L Hallas J Land L H Herrstedt J Comorbidity and polypharmacy in elderly cancer patients: the significance on treatment outcome and tolerance J Geriatr Oncol 2010 1 02 87 102 10.1016/j.jgo.2010.06.002

[22] Food and Drug Administration CFR – Code of Federal Regulations Title 21: Investigational New Drug Application2024

[23] Cancer Institute N Common Terminology Criteria for Adverse Events (CTCAE) v5.02017 https://www.meddra.org/

[24] Sterne J AC Hernán M A Reeves B C ROBINS-I: a tool for assessing risk of bias in non-randomised studies of interventions BMJ 2016 355 i4919 10.1136/bmj.i4919 27733354 PMC5062054

[25] Newcastle-Ottawa Quality Assessment Form for Cohort Studies

[26] McMaster University and Evidence Prime GRADEpro GDT: GRADEpro Guideline Development Tool [Software]2024

[27] Ikeda A Hoshi N Yoshizaki T Endoscopic submucosal dissection (ESD) with additional therapy for superficial Esophageal cancer with submucosal invasion Intern Med 2015 54 22 2803 2813 26567992 10.2169/internalmedicine.54.3591

[28] Koterazawa Y Nakamura T Oshikiri T A comparison of the clinical outcomes of Esophagectomy and Chemoradiotherapy after noncurative endoscopic submucosal dissection for Esophageal squamous cell carcinoma Surg Today 2018 48 08 783 789 10.1007/s00595-018-1650-y 29532261 PMC6060875

[29] Tanaka T Ueno M Iizuka T Hoteya S Haruta S Udagawa H Comparison of long-term outcomes between Esophagectomy and Chemoradiotherapy after endoscopic resection of submucosal Esophageal squamous cell carcinoma Dis Esophagus 2019 32 12 doz023 10.1093/dote/doz023 30980070

[30] Suzuki G Yamazaki H Aibe N Chemoradiation versus surgery for superficial Esophageal squamous cell carcinoma after noncurative endoscopic submucosal dissection: comparison of long-term oncologic outcomes Radiat Oncol 2022 17 01 191 10.1186/s13014-022-02162-8 36401267 PMC9675257

[31] Li J Wang L Shi X Therapeutic strategies following endoscopic submucosal dissection for T1a-MM/T1b-SM1 Esophageal squamous cell carcinoma: comparisons of long-term outcomes with propensity score-matched analysis Surg Endosc 2023 37 03 1761 1770 10.1007/s00464-022-09683-z 36220991

[32] Xiao L Ren L Qiu Y Yang C Shi R Efficacy of additional treatment after endoscopic submucosal dissection for superficial Esophageal squamous cell carcinoma Chin J Dig Endosc 2022 118 122

[33] Zhu K Sun Y Liu Z Adjuvant strategies for patients with T1b invasion after endoscopic submucosal dissection for Esophageal squamous cell carcinoma Shanghai Jiao Tong Da Xue Xue Bao 2024 116 123

[34] Hisano O Nonoshita T Hirata H Additional radiotherapy following endoscopic submucosal dissection for T1a-MM/T1b-SM Esophageal squamous cell carcinoma improves Locoregional control Radiat Oncol 2018 13 14 10.1186/s13014-018-0960-y 29378603 PMC5789550

[35] Lee H J Lee H Park J C Shin S K Lee S K Lee Y C Treatment strategy after endoscopic resection of superficial Esophageal squamous cell carcinoma: a single institution experience Gut Liver 2015 9 06 714 719 10.5009/gnl14142 25473076 PMC4625699

[36] Dirr V Vetter D Sartoretti T Schneider M A Da Canal F Gutschow C A Quality of life and independent factors associated with poor digestive function after Ivor Lewis Esophagectomy Cancer 2023 15 23 5569 10.3390/cancers15235569 PMC10705190 38067274

[37] Tae C H Shim K N Kim B W Comparison of subjective quality of life after endoscopic submucosal resection or surgery for early gastric cancer Sci Rep 2020 10 01 6680 10.1038/s41598-020-62854-7 32317659 PMC7174391

[38] Jezerskyte E van Berge Henegouwen M I van Laarhoven H WM Postoperative complications and long-term quality of life after multimodality treatment for Esophageal cancer: an analysis of the prospective observational cohort study of Esophageal-gastric cancer patients (POCOP) Ann Surg Oncol 2021 28 12 7259 7276 10.1245/s10434-021-10144-5 34036429 PMC8519926

[39] Minashi K Nihei K Mizusawa J Efficacy of endoscopic resection and selective Chemoradiotherapy for stage I Esophageal squamous cell carcinoma Gastroenterology 2019 157 02 382 390000 10.1053/j.gastro.2019.04.017Epub 2019 Apr 20. PMID: 31014996 31014996

[40] Mochizuki Y Saito Y Tsujikawa T Fujiyama Y Andoh A Combination of endoscopic submucosal dissection and Chemoradiation therapy for superficial Esophageal squamous cell carcinoma with submucosal invasion Exp Ther Med 2011 2 06 1065 1068 10.3892/etm.2011.319 22977621 PMC3440818

[41] Yamamoto Y Ishihara R Kawakubo H Comparison of outcomes between surgery and Chemoradiotherapy after endoscopic resection for pT1a-MM with Lymphovascular invasion or pT1b Esophageal squamous cell carcinoma: Japanese Multicenter propensity score-matched study J Gastroenterol 2025 60 01 43 54 10.1007/s00535-024-02188-7 39625653 PMC11717814

