# Supplementary material for: Endoscopic Submucosal Dissection and Chemoradiotherapy versus Esophagectomy for T1a-m3 or T1b Esophageal Squamous Cell Carcinoma: A Meta-analysis
Source: Endosc Int Open. 2026 Aug 14;14:a29264315. doi: 10.1055/a-2926-4315 (PMC13492310; doi:10.1055/a-2926-4315)
Supplement: Supplementary file 1 [file 10-1055-a-2926-4315_29315157.pdf]

Endoscopic submucosal dissection with adjuvant chemoradiotherapy versus esophagectomy for T1aM3 or T1b esophageal squamous cell carcinoma: a systematic review and meta-analysis

Table S1. Search strategy

Database	Search query
**Medline**	("oesophagus neoplasm"[All Fields] OR "esophageal neoplasms"[MeSH Terms] OR ("esophageal"[All Fields] AND "neoplasms"[All Fields]) OR "esophageal neoplasms"[All Fields] OR ("esophagus"[All Fields] AND "neoplasm"[All Fields]) OR "esophagus neoplasm"[All Fields] OR ("esophageal squamous cell carcinoma"[MeSH Terms] OR ("esophageal"[All Fields] AND "squamous"[All Fields] AND "cell"[All Fields] AND "carcinoma"[All Fields]) OR "esophageal squamous cell carcinoma"[All Fields])) AND ((("endoscope s"[All Fields] OR "endoscoped"[All Fields] OR "endoscopes"[MeSH Terms] OR "endoscopes"[All Fields] OR "endoscope"[All Fields] OR "endoscopical"[All Fields] OR "endoscopically"[All Fields] OR "endoscopy"[MeSH Terms] OR "endoscopy"[All Fields] OR "endoscopic"[All Fields]) AND ("dissect"[All Fields] OR "dissected"[All Fields] OR "dissecting"[All Fields] OR "dissection"[MeSH Terms] OR "dissection"[All Fields] OR "dissections"[All Fields] OR "dissects"[All Fields])) OR (("endoscope s"[All Fields] OR "endoscoped"[All Fields] OR "endoscopes"[MeSH Terms] OR "endoscopes"[All Fields] OR "endoscope"[All Fields] OR "endoscopical"[All Fields] OR "endoscopically"[All Fields] OR "endoscopy"[MeSH Terms] OR "endoscopy"[All Fields] OR "endoscopic"[All Fields]) AND ("resect"[All Fields] OR "resectability"[All Fields] OR "resectable"[All Fields] OR "resectates"[All Fields] OR "resected"[All Fields] OR "resecting"[All Fields] OR "resection"[All Fields] OR "resectional"[All Fields] OR "resectioned"[All Fields] OR "resectioning"[All Fields] OR "resections"[All Fields] OR "resective"[All Fields] OR "resects"[All Fields])) OR ("earth syst dyn"[Journal] OR "esd"[All Fields]) OR ("mucosectomies"[All Fields] OR "mucosectomy"[All Fields])) AND ("neoadjuvant therapy"[MeSH Terms] OR ("neoadjuvant"[All Fields] AND "therapy"[All Fields]) OR "neoadjuvant therapy"[All Fields] OR (("adjuvancy"[All Fields] OR "adjuvanted"[All Fields] OR "adjuvanting"[All Fields] OR "adjuvants"[All Fields] OR "adjuvants pharmaceutic"[Pharmacological Action] OR "adjuvants immunologic"[Pharmacological Action] OR "adjuvants, pharmaceutic"[MeSH Terms] OR ("adjuvants"[All Fields] AND "pharmaceutic"[All Fields]) OR "pharmaceutic adjuvants"[All Fields] OR "adjuvant"[All Fields] OR "adjuvants, immunologic"[MeSH Terms] OR ("adjuvants"[All Fields] AND "immunologic"[All Fields]) OR "immunologic adjuvants"[All Fields] OR "adjuvated"[All Fields] OR "adjuvation"[All Fields] OR "adjuvent"[All Fields]) AND ("therapeutics"[MeSH Terms] OR "therapeutics"[All Fields] OR "therapies"[All Fields] OR "therapy"[MeSH Subheading] OR "therapy"[All Fields] OR "therapy s"[All Fields] OR "therapys"[All Fields])) OR ("chemotherapy s"[All Fields] OR "drug therapy"[MeSH Terms] OR ("drug"[All Fields] AND "therapy"[All Fields]) OR "drug therapy"[All Fields] OR "chemotherapies"[All Fields] OR "drug therapy"[MeSH Subheading] OR "chemotherapy"[All Fields]))
**Google Scholar**	esophageal squamous cell carcinoma OR esophagus neoplasm OR esophagus cancer "T1a OR T1b OR superficial OR early" "endoscopic submucosal dissection" "chemoradiotherapy OR radiotherapy OR chemotherapy" "esophagectomy"
**Embase**	('esophagus tumor'/exp OR 'esophagus tumor' OR 'esophagus cancer'/exp OR 'esophagus cancer') AND ('endoscopic submucosal dissection'/exp OR 'endoscopic submucosal dissection') AND ('chemotherapy'/exp OR chemotherapy OR 'radiotherapy'/exp OR radiotherapy OR 'adjuvant therapy'/exp OR 'adjuvant therapy' OR 'chemoradiotherapy'/exp OR chemoradiotherapy)

Table S1. *Search strategy used to query the electronic databases (Medline, Google Scholar, and Embase), combining keywords and MeSH terms related to esophageal neoplasms, endoscopic submucosal dissection (ESD), chemoradiotherapy, and esophagectomy.*

Table S2. Risk-of-bias assessment (ROBINS-I)

Study	Confounding	Selection bias	Bias in the evaluation of interventions	Bias due to deviations from interventions	Bias due to missing data	Bias in outcomes	Bias in reported results	Overall
Yasufumi Koterazawa	Low	Low	Low	Low	Low	Low	Low	Low
Tsuyoshi Tanaka	Low	Low	Low	Low	Low	Low	Low	Low
Gen Suzuki	Moderate	Moderate	Moderate	Low	Low	Low	Low	Moderate
Jun Li	Moderate	Low	Moderate	Low	Moderate	Low	Low	Moderate
Xiao Li	Moderate	Serious	Moderate	Low	Low	Critical	Low	Critical
ZHU Kaiyuan	Moderate	Serious	Low	Low	Low	Moderate	Low	Serious

Table S2. *Risk-of-bias assessment performed with the ROBINS-I tool. Yasufumi Koterazawa and Tsuyoshi Tanaka showed minimal bias, indicating high methodological quality, whereas Xiao Li and ZHU Kaiyuan carried the most substantial risk of bias.*

Table S3. Risk-of-bias assessment (Newcastle–Ottawa Scale)

Study	Representativeness of ESD + CRT (0–1)	Selection of esophagectomy (0–1)	Diagnostic evaluation (0–1)	Outcome of interest absent at study initiation (0–1)	Comparability of cohorts by design or analysis (0–2)	Outcome assessment (0–1)	Duration of follow-up (0–1)	Adequacy of cohort follow-up (0–1)	AHRQ standard
Yasufumi Koterazawa	1	1	1	1	2	1	1	1	Good
Tsuyoshi Tanaka	1	1	1	1	1	1	1	1	Good
Gen Suzuki	1	1	1	1	1	1	0	1	Good
Jun Li	0	1	1	1	1	0	1	1	Good
Xiao Li	0	1	1	0	0	1	1	1	Poor
ZHU Kaiyuan	0	1	1	1	1	1	0	1	Fair

Table S3. *Risk-of-bias assessment performed with the Newcastle–Ottawa Scale (NOS). Representativeness of the ESD + CRT cohort and diagnostic evaluation were the most common areas of weakness among the lower-rated studies.*

Table S4. Certainty of evidence (GRADE)

Outcome (follow-up)	No. of studies	Study design	Risk of bias	Inconsistency	Indirectness	Imprecision	Other	ESD + CRT	Esophagectomy	Relative effect (95% CI)	Certainty	Importance
**Adverse events (mean follow-up 47 months)**	4	Observational	Not serious	Not serious	Not serious	Not serious	None	28/118	30/84	**RD −0.15 (−0.28 to −0.02)**	⊕⊕⊕⊕ High	Important
**3-year overall survival (mean follow-up 44.5 months)**	7	Observational	Not serious	Not serious	Not serious	Not serious	None	168/181	141/151	**RD −0.08 (−0.15 to −0.01)**	⊕⊕⊕⊕ High	Critical
**5-year overall survival (mean follow-up 55.2 months)**	4	Observational	Not serious	Not serious	Not serious	Not serious	None	100/117	79/87	**RD −0.09 (−0.18 to 0.01)**	⊕⊕⊕⊕ High	Critical
**3-year disease-free survival (mean follow-up 44.5 months)**	7	Observational	Not serious	Not serious	Not serious	Not serious	None	155/181	139/151	**RD −0.08 (−0.15 to −0.01)**	⊕⊕⊕⊕ High	Important

Table S4. *GRADE summary-of-findings table. High-certainty evidence (⊕⊕⊕⊕) indicates that ESD + CRT and esophagectomy have comparable outcomes for adverse events, 3-year and 5-year overall survival, and 3-year disease-free survival, with a slight benefit favouring ESD + CRT. All outcomes were rated important or critical. The ESD + CRT and esophagectomy columns show the number of events / total patients (n/N); "Other" denotes other considerations. RD, risk difference; CI, confidence interval.*

Abbreviations: ESD, endoscopic submucosal dissection; CRT, chemoradiotherapy; OS, overall survival; DFS, disease-free survival; ROBINS-I, Risk Of Bias In Non-randomised Studies of Interventions; NOS, Newcastle–Ottawa Scale; AHRQ, Agency for Healthcare Research and Quality; GRADE, Grading of Recommendations Assessment, Development and Evaluation.
